# Surviving under pressure

**DOI:** 10.7554/eLife.90216

**Published:** 2023-07-12

**Authors:** Ying Wang, Liandong Yang

**Affiliations:** 1 Hubei Engineering Research Center for Protection and Utilization of Special Biological Resources in the Hanjiang River Basin, College of Life Sciences, Jianghan University Wuhan China; 2 Liandong Yang is at the State Key Laboratory of Freshwater Ecology and Biotechnology, Institute of Hydrobiology, Chinese Academy of Sciences, Wuhan, China Beijing China

**Keywords:** hadal snailfish, Tanaka's snailfish, adaptation, extreme environments, hadal zone, Other

## Abstract

Genomic analysis has shed light on how hadal snailfish have adapted to living at depths of several thousand metres.

**Related research article** Xu WJ, Zhu C, Gao X, Wu B, Xu H, Hu ML, Zeng H, Gan X, Feng CG, Zheng J, Bo J, He L, Qiu Q, Wang W, He S, Wang K. 2023. Chromosome-level genome assembly of hadal snailfish reveals mechanisms of deep-sea adaptation in vertebrates. *eLife* **12**:RP87198. doi: 10.7554/eLife.87198.

The world’s oceans are divided into five depth zones, with the hadal zone – which refers to depths of more than 6000 metres – being the deepest. Composed mainly of deep trenches, the hadal zone is among the most hostile environments on Earth because it is extremely cold and dark, there is very little food, the trenches are geographically isolated, and the hydrostatic pressure can reach values as high as 1000 times atmospheric pressure (Somero, 1992; Jamieson, 2015).

The most common vertebrate species in the hadal zone are fish called snailfishes, and hadal snailfishes can survive down to depths of about 8100 metres (Linley et al., 2016). Other species of snailfish live in coastal waters, which means that the snailfish (sometimes known as the sea snail) has the widest depth range of any marine fish species. Researchers have identified various ways in which hadal snailfish have adapted to their extreme environment (Wang et al., 2019; Mu et al., 2021), but we still do not fully understand how snailfish evolved and why they are among the few vertebrate species that have successfully adapted to the hadal zone.

Now, in eLife, Shunping He (Institute of Hydrobiology, Chinese Academy of Sciences), Kun Wang (Northwestern Polytechnical University) and colleagues – including Wenjie Xu, Chenglong Zhu, Xueli Gao, Baosheng Wu, Han Xu, Mingliang Hu and Honghui Zeng as joint first authors – report the results of a genomic study that provides new insights into the origin and evolution of the hadal snailfish (Xu et al., 2023).

Xu et al. started by generating genomic data for four hadal snailfish that had been collected from the Mariana Trench in the Northwest Pacific Ocean, and four Tanaka’s snailfish that had been collected from the Southern Yellow Sea: Tanaka’s snailfish is a close relative of the hadal snailfish that lives in shallower waters. After a series of thorough bioinformatic analyses, they identified 33 genes that are only found in hadal snailfish, 19 unitary pseudogenes, and various other differences between hadal snailfish and related species. For instance, there are 21 genes for which the gene number in hadal snailfish is higher than the gene number in Tanaka’s snailfish. Strikingly, most of these genes and differences had not been observed before, probably due to the fragmented nature of early versions of the hadal snailfish genome. Xu et al. also observed that 51 genes present in other snailfish are not present in Hadal snailfish.

By comparing genomic and mitochondrial data belonging to snailfishes from different trenches – including the Kermadec Trench, which is about 6,400 kilometres from the Mariana Trench – they found that hadal snailfishes have successfully spread to multiple trenches in the Pacific Ocean over the course of a million years.

By associating gene variation, expression, and function, Xu et al. were able to yield several important insights into the ways the hadal snailfish has adapted to its extreme environment (Figure 1). First, the *rh1* gene, which is critical for monochromatic vision in very dim light, is present and expressed in hadal snailfish, whereas other genes that are associated with seeing at different wavelengths (*lws, rh2* and *sws2*) are lost or hardly expressed.

**Figure 1.**
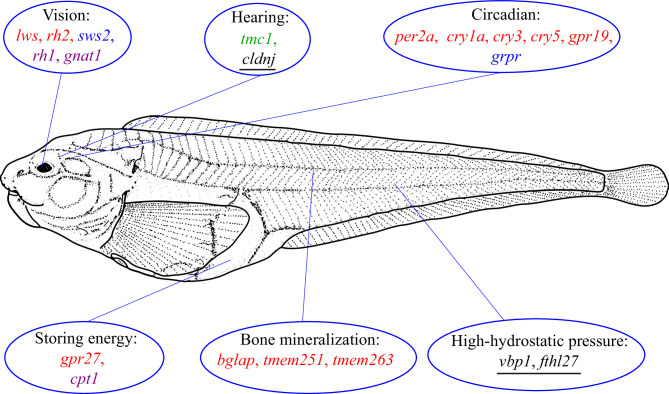
How snailfish adapt to the hadal zone. The genome of the hadal snailfish differs from the genomes of other snailfish in a number of ways that help it adapt to life in the extreme environment of the hadal zone. Genes for monochromatic vision in very dim light (*rh1* and *gnat1*) are present and expressed in hadal snailfish, whereas genes for seeing at long (lws) and central wavelengths (*rh2*) are lost, and a gene for seeing at short wavelengths (*sws2*) is present but barely expressed. Hadal snailfish also have three copies of *cldnj*, a gene associated with hearing, while another auditory gene (*tmc1*) is upregulated. Various circadian rhythm genes are either absent (*per2a*, *cry1a*, *cry3*, *cry5* and *gpr19*), or are present but barely expressed (*grpr*); however, a small number of essential circadian clock control genes are present and expressed. A gene called *gpr27* that is involved in metabolism has become a pseudogene, whereas another metabolic gene (*cpt1*) is upregulated. Three genes related to bone mineralization (*bglap*, *tmem251*, and *tmem263*) have been lost, whereas there are multiple copies of two genes that help hadal snailfish cope with high hydrostatic pressures (fthl27 [14 copies] and vbp1 [two copies]). Red text: genes are absent in the hadal snailfish; blue text: genes are present but barely expressed; violet text: genes are present and are expressed; green text: genes are present and are upregulated; underlined text: multiple copies are present.

Second, the majority of the auditory genes were preserved in hadal snailfish and many of them were upregulated, probably to compensate for the loss of visual genes. Third, while many circadian rhythm genes have been lost, or have become pseudogenes, a small number of essential circadian clock control genes are present and expressed in the hadal snailfish, indicating that a rhythm cycle is retained, although it is probably not coupled to the day-night cycle.

Fourth, a gene called *gpr27* that is involved in metabolism in other species is a pseudogene in the hadal snailfish, which probably helps it to reduce metabolism and store energy in order to survive periods when food is not available. Fifth, two genes that are involved in bone mineralization in other snailfish are not found in hadal snailfish: the fact that the skull of the hadal snailfish is not completely enclosed allows for the equalization of the internal and external pressure.

Finally, there are 14 copies of a gene called *fthl27* in hadal snailfish, compared with just three copies in Tanaka’s snailfish. This gene encodes a protein called ferritin, and Xu et al. suggest that the overexpression of this gene may increase the tolerance of cells to the high levels of reactive oxygen species that are found at high hydrostatic pressures: these pressures can disrupt cellular processes and cause oxidative stress, resulting in the production of reactive oxygen species.

Our understanding of the genetic basis of adaptation to the hadal zone continues to improve, thanks to the work of Xu et al. and other researchers. However, many questions remain unanswered. An important next step will be to perform experiments in the lab – as Xu et al. have done – in order to validate (or otherwise) what comparative genomics studies are suggesting. We just have to look.
